# Why geographic data science is not a science

**DOI:** 10.1111/gec3.12537

**Published:** 2020-07-24

**Authors:** Simon Scheider, Enkhbold Nyamsuren, Han Kruiger, Haiqi Xu

**Affiliations:** ^1^ Department of Human Geography and Spatial Planning Utrecht University The Netherlands

**Keywords:** community of practice, geographic data science, Geography, GIScience, meta‐science, scientific concepts, scientific questions

## Abstract

“Data Science” has taken many disciplines by storm. And for a good reason: New forms and unseen quantities of data enter nearly every scientific field, substantially changing the ways how scientists do science, and potentially allowing them to answer old questions or to pose them in novel ways. The recent success of Data Science is also reflected in corresponding study programs and curricula and the emergence of specialized branches, such as Geographic Data Science (GDS). Some researchers, therefore, claim that Data Science and GDS should be treated as autonomous scientific disciplines, while others fear that it sells nothing but old wine in new bottles. In an attempt to sober the discussion, we investigate GDS and Data Science from the perspective of meta‐science. We provide arguments why today's GDS and Data Science should be seen as an interdisciplinary community of practice of data‐driven scientists, rather than a scientific discipline. We also discuss what is missing for GDS and Data Science to become genuine scientific disciplines.

## 1 INTRODUCTION AND CONTEXT

There is a wave of recent publications and curricula adoptions concerning *Data Science* within various disciplines, from Geosciences, Biology (Kyrpides, Eloe‐Fadrosh, & Ivanova, 2016), over Health Science to Sociology and Human Geography (Kitchin, 2014; Loukides, 2011; Schutt & O'Neil, 2013). In the wake of these developments, a discussion has been started about what this “data revolution” really entails for these disciplines. There is no doubt that we are facing a paradigm shift in the way we do science since the ever‐increasing importance of volume, velocity, variety, and veracity (quality) of data in these disciplines asks for new methods to do science in a “data‐intensive” way.

This revolution towards the so‐called fourth paradigm (Hey, Tansley, Tolle, 2009) is also visible in Geography, Geosciences, and GIScience (Kitchin, 2013), for example, in the adoption of a data‐driven and artificial intelligence (AI) fuelled approach to capturing the diverse processes in a “smart” city (Batty, 2013) with computational tools (Degbelo et al., 2016). This development has recently spurred calls for a new autonomous discipline, called *Geographic Data Science (GDS)* (Arribas‐Bel & Reades, 2018), which is supposed to be a new science about the relationship between Geography and computers (see also several blogs about this issues here^1^ and here^2^).

The argument of Arribas‐Bel and Reades (2018), in summary, goes like this: What (quantitative) geographers have been doing in the past (starting from the quantitative revolution [Barnes, 2014])—including data gathering, preparation, and exploration; data representation and transformation; computing with data; data visualization and presentation; data modeling, as well as reflecting about these methods—belongs to the core competences of a modern age data scientist (Donoho, 2017). Furthermore, the new data sources require revolutionary ways to handle data (e.g., personal data, ubiquitous sensors, and computing resources), and there is a need to incorporate ideas and practices that are neither part of the scientific domain (e.g., Geography) nor part of the methodology (e.g., Statistics, Machine Learning). So why not situate such a discipline in the new modern context of data science?

[…] data science provides a framework to not only better understand, but also to effectively leverage, the kind of broadly defined “data” that is of interest to geographers. (Arribas‐Bel and Reades (2018))

In arguing for Geographic Data Science, Arribas‐Bel et al. seem to regard Geographic Information Science (*GIScience*)^3^ as kind of synonymous to the new discipline, or even as one of its essential parts. In a similar vein, Luc Anselin recently expressed the view that “GIScience [is] morphing into spatial data science.”^4^ In this view, (quantitative) Geography as well as any kind of geographic information science slowly but inevitably dissolves into being just one of the many “data sciences” dealing with geographic information. From an engineering perspective, Raubal (2019) recently argued to regard spatial data science as a more interdisciplinary and thus broader version of GIScience.

In this discussion article, we argue not only why the view of seeing GIScience as part of Geographic Data Science is fundamentally misleading, but also why Geographic Data Science, at least in its current state, can hardly be considered a distinct scientific discipline on its own. In particular, we argue in this paper that:

- Geographic Data Science is currently a *community of practice* of (data‐driven) Geography or Geoscience, and therefore not (yet) a distinct scientific discipline.
- GIScience, in contrast, is a distinct *meta‐scientific* discipline, that is, a discipline *about (geographic information) methods*. In a nutshell, GIScientists are not Geographers in the same sense that Statisticians are not called Biologists, even when developing methods for Biology.
- GIScience is therefore *not* a subset of Geographic Data Science, for the same reason it is not subsumed by Geography or any other Geoscience.
- To become a scientific discipline of its own, we think Geographic Data Science needs to move up to a meta‐science level. This means it needs to develop its own *science about methods*.
- For this purpose, Geographic Data Science first and foremost needs to develop its own questions and concepts, distinguishing it from other disciplines such as GIScience, Statistics or Computer Science.
- However, currently, we fail to see which concepts these might be, and which are not already covered by these other disciplines.

The distinctions suggested here are essential for several reasons. For one, the *status of a science* not only implies own academic resources, it also implies academic legitimacy in graduate level teaching and in corresponding distinguishable research. Similar to GIScience some decades ago (Wright, Goodchild, & Proctor, 1997), if GDS is to become a scientific discipline, it will legitimately reach beyond a mere “technical support” role.

But also content‐wise, our distinctions help clarify what is at stake. The current tendency to intermingle terms comes namely with a *confusion of research goals*. If GDS is only considered a re‐branding of GIScience or Geography in the data‐intensive era, then the term degenerates into a predictable marketing manoeuvre, without any genuine new goals. Note that both Geography (Kitchin, 2014) and GIScience (Gahegan, 2020) are actively using and also further developing data‐intensive methods. If, on the other hand, it is seen as a genuine data‐driven substitution of GIScience, then 40 years of *meta‐scientific* research are at stake, especially research that is *not* purely data driven (cf. the discussion in Section 4). In a nutshell, our argument is that, since information is more than data, data driven methods are not sufficient for dealing with purpose and other essential information concepts on the meta‐level. Similarly, Geography would loose a lot when substituted by GDS, as illustrated by the old but ongoing discussion about the quantitative/qualitative divide (Barnes, 2014).

In what follows, we not only explain these distinctions in greater detail, but also investigate what exactly it would take for Geographic Data Science to become a proper discipline on its own, and we make some suggestions in that respect. We start by arguing what is required to be called a discipline in general and a meta‐science discipline in particular.

## 2 RELATED CONCEPTS

The question is whether Geographical Data Science (GDS) can be considered a discipline distinct from other scientific disciplines. To this end, we need to localize GDS with respect to the triangle spanned by Data Science, Geographical Information Science (GIScience) and Geography. As a science, GIScience however operates on a different level. To fully comprehend the potential of GDS as an independent discipline, we therefore need to look at GDS via the prism of meta‐science as well. In the following, we first discuss requirements of a scientific discipline, and then refine the term meta‐science, which refers to a subgroup of disciplines concerned with scientific methods.

### 2.1 Scientific disciplines versus communities of practice

Within scientific research, we distinguish many different scientific disciplines, such as Physics, Mathematics, Biology, Geography, Information Science, and so on. Scientists from a particular discipline usually not only share a common view on the subject of their research, they also share the means to do their research, including general scientific standards of argumentation, analysis, and testing. In empirical disciplines such as Geography, this means researchers adopt standards of statistical or qualitative testing (Lindsay, 2006). In Information Science (Iivari, 2005), which deals with the design of information products, requirements and rigor cycles ensure the quality of the design instead (Hevner, 2007). However, in all cases, these disciplines form *communities of practice*, which have a specific interest in common, and which share methods, standards, and practices:

Claim 1 Every scientific discipline forms a community of practice.

For example, many geographers have intimate experience with using clustering methods. The same can be said about psychologists. Thus, each of these researcher groups may form a community of practice of clustering. However, do geographers and psychologists therefore together form a distinct discipline about clustering methods? We think not, because they operate with different concepts and questions to which clustering is applied. Hence, we argue that the converse is not true:

Claim 2 Not every community of practice is a scientific discipline.

Being a discipline requires to the very least (as a necessary condition) to be distinguishable not only in terms of scientific practices and interests but also *in terms of concepts and questions* (cf. Chalmers, 2013):

Claim 3 A scientific discipline requires a distinguishing set of questions about its own set of concepts.

A scientific discipline is a community of practice that can be distinguished based on its own concepts and the kinds of questions that are asked about these concepts. For example, physicists have a concept of particles, and they ask distinctive questions about their behavior and their effects on larger‐scale phenomena. Environmental health scientists have a concept of exposure, and they study questions about the effect of exposure on the health of individuals (Rappaport, 2011). Human geographers have a concept of place, and how places evolve in time and how they, in turn, influence the lives of people (Johnston, 1991). Mathematicians have a concept of sets, and they study the behavior of different kinds of sets under different kinds of operations or algebras (e.g., partial orders, rings, metric spaces, …; Stell & Worboys, 1997). Clearly, a discipline can be distinguished by the types of questions, and the questions can be distinguished by the types of concepts they are about.

### 2.2 Empirical versus meta‐science

In modern science, there is an important division to be made between disciplines that seek to apply scientific practices to empirical domains of study on the one hand, and disciplines that seek to develop and improve scientific practices, a.k.a. meta‐science disciplines, on the other hand. The former seek to explore and explain objects, events, and other phenomena measured and experienced in the world. For this purpose, they gather data, use measurement frameworks, methodological frameworks, and analytical tools to propose hypotheses and establish theories. Furthermore, they run models about the phenomenon of study. All this applies, for example, to Geography. In contrast, a meta‐science discipline is about the methodology of science. It is basically about *design* (Wieringa, 2010): It explores how data, methodological frameworks, and analytical tools can be used to infer hypotheses and theories, and why it is valid to do so and for which purpose. At its core, a meta‐science discipline develops new methods based on theory, and new concepts and new theories about already existing methods. It, therefore, goes beyond “problem solving” or engineering, see Wright et al. (1997). In the remainder of this article, we use the term *empirical discipline*, in contrast to a *meta‐science discipline*, to refer to any discipline that explores a particular domain of experience.

Very successful examples of meta‐science disciplines are Statistics, Machine Learning, and Information Science. For instance, Couclelis (2016) argues for seeing *Information Science* (Iivari, 2005) as a meta‐science discipline, which is especially relevant in our context:

It may be useful to distinguish clearly between the empirical sciences that directly measure and represent phenomena in the world, and the information sciences (which are meta‐sciences) that process and present information about these phenomena in ways that meet and support the interests and purposes of information users. These are two different epistemic layers with different functions. I will argue for the importance of not conflating the two because as information scientists we are not doing Hydrology, Forestry or Urban Studies but trying to help answer questions posed by hydrologists, foresters, planners, and any others, in the most appropriate and helpful ways. (Couclelis, 2016)

The objects of study in Information Science are not empirical phenomena themselves (such as a river or a city), but the different sorts of information that can be obtained about them. This includes how phenomena can best be measured, represented and analyzed for a given purpose, and what kind of knowledge one can infer from them. For example, Information Science may study what precision and accuracy of data are (Devillers, Jeansoulin, & Goodchild, 2006), how measurement scales can influence data quality (Chrisman, 1998), or how a causal relationship can be detected in data (Pearl & Mackenzie, 2018). It may also investigate how efficient a certain method is or how fit data is for a given purpose (Brown et al., 2013). Questions and answers are about concepts that relate to the methods and representations used in the underlying discipline, and for a particular purpose which relates to this discipline. For example, “What is the best estimator of spatial dependency?” (Cressie, 1988) or “What is the best method to assess a line of sight?” (Fisher, 1993) are questions of GIScience closely related to the purposes of spatial interpolation and viewshed analysis in the geosciences. *Purpose* and *intention*, therefore, play an central role in meta‐science (Couclelis, 2010), as well as *measurement*, *perception*, and *selection* of information (Scheider, Ostermann, & Adams, 2017). Whenever an urban researcher, for instance, selects a sample of tweets to collect data for disaster management, information scientists may warn him to make sure the sample is not biased towards certain types of user groups (Granell & Ostermann, 2016). This is the reason why information theory needs to be based on pragmatics, measurement theory, or cognition; rather than on Physics (Couclelis, 1992, 1997, 2009, 2016). Most importantly, however, just because it is on the meta‐level, the work of information scientists should not be considered in any way “less scientific” than the work of colleagues in the respective empirical disciplines (Wright et al., 1997). Meta‐science is *full blown* science, both concerning theory development (e.g., about conceptual models) as well as empirical methods (e.g., in user or benchmark studies).

It is thereby not important whether methods and questions of empirical and meta‐science disciplines neatly map onto each other. We can have questions for which no method exists yet, and we may have novel methods for which the questions are still unclear. Furthermore, methods also do not need to be *data‐driven*. Meta‐scientific methods are often *not data driven*.^5^ For example, Statistics and Machine Learning are related yet distinct meta‐science disciplines with their own set of concepts, including for example *hypothesis tests* versus *cross‐validation* (Cudeck & Browne, 1983). To answer their questions, both disciplines thereby mainly use methods *other* than the ones they produce: Statisticians do not use statistical tests to design a new statistical test, and machine‐learning algorithms are not designed using machine‐learning algorithms.^6^ Rather, these methods are designed based on their own theories.

And here comes an important implication: It is for good reasons that a scientist who practices statistical methods cannot be called a statistician. If a biologist uses a regression model to investigate a biological system, it does not make the biologist a statistician. In the same sense, a geographic data scientist using a buffer does not become a GIScientist. This highlights that the research questions are located on totally different levels: Researchers engaging in meta‐science ask questions on a higher level than their colleagues in the corresponding empirical discipline. A Geographer asks about a city, while a GIScientist asks about the ways of representing a city via spatial concepts. Of course, this does not mean that the same scientist may sometimes “change hats” and redirect questions from the empirical level to the method level. In this way, the Modifiable Area Unit Problem (MAUP), for example, originated in Geography and made its way into GIScience (Openshaw, 1981). However, concerning goals, the work of meta‐scientists is on a different level. As scientists, they pursue different goals.

## 3 WHY DATA SCIENCE IS NOT A SCIENTIFIC DISCIPLINE

We believe it is mainly a confusion of goals that makes it difficult to understand the role of Data Science. The best example of this confusion is the term “Data Science” itself, which implies a science *of data* (i.e., where the object of research is data), while it occurs to be nothing more than a community of scientists *using data in a certain way*. In this section, we argue why data science in its current state should rather not be seen as a discipline on its own, but rather as a community of practice. For this purpose, we first review the definitions of Data Science, before we argue that Data Science should be regarded as the latter rather than the former.

### 3.1 What is data science?

In its earliest mentions by Naur (1974), Data Science was seen as a substitute for Computer Science or “Datalogy.” Since the 1990s, Data Science reappeared as a term. Since then, it was rather seen from the viewpoint of Statistics, as by C.F. Jeff Wu.^7^ Let us thus look at some common contemporary definitions of Data Science.

Data Science is the extraction of knowledge from data.^8^

This definition^9^ may well describe the practice of data scientists, which largely lies in applying methods from Computer Science, Statistics, and Machine Learning to gain knowledge inside some empirical discipline. Unfortunately, it does not distinguish Data Science from a lot of other empirical disciplines, such as Physics, Chemistry, Psychology, and so on which use the same methods and questions, and in general from Knowledge Discovery in Databases (Fayyad, Piatetsky‐Shapiro, & Smyth, 1996; Frawley, Piatetsky‐Shapiro, & Matheus, 1992; Piatetsky‐Shapiro & Frawley, 1991)^10^:

Knowledge Discovery in Databases (KDD) is the process of discovering useful knowledge from a collection of data.

**FIGURE 1:**
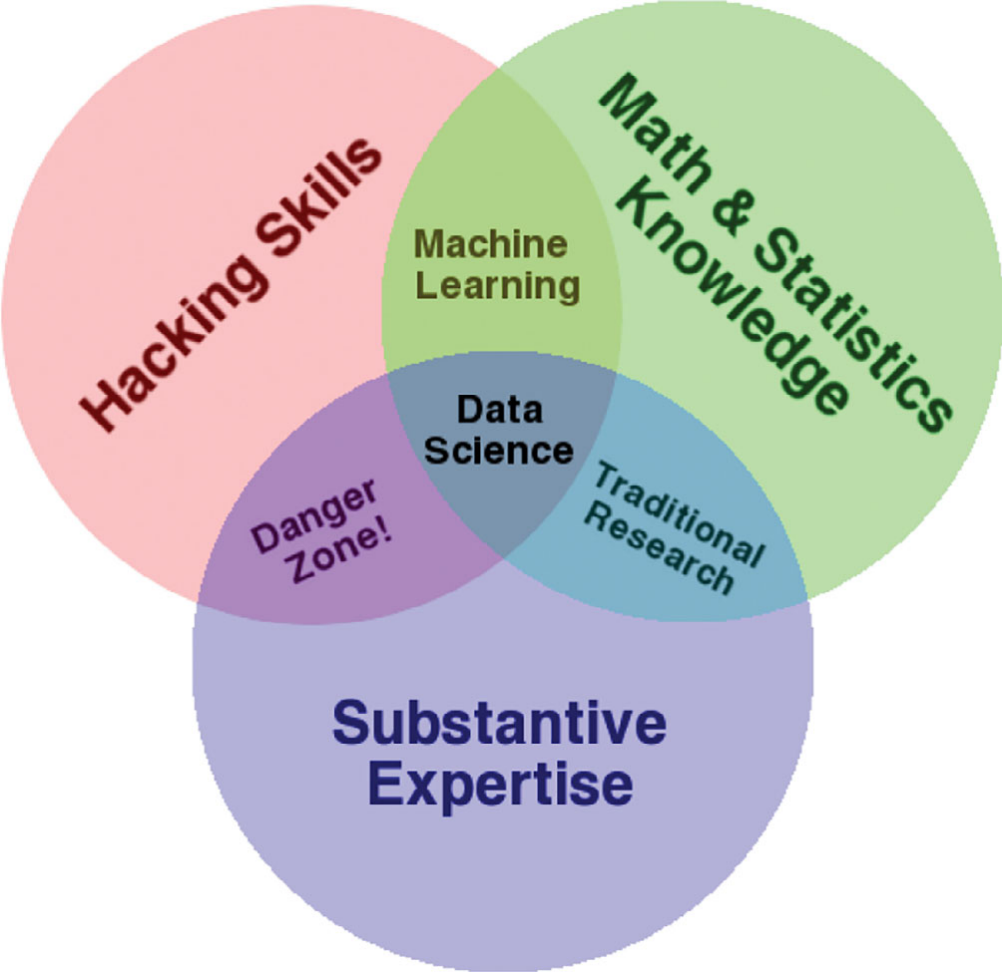
Definition of data science by Drew Conway (drewconway.com/zia/2013/3/26/the‐data‐science‐venn‐diagram)

Still, we may grant that data scientists do stuff differently than their KDD predecessors in the 1990s. Drew Conway, a prominent data scientist, therefore, proposed to regard Data Science as an intersection of at least three skills, namely domain knowledge (substantive expertise), “hacking skills,” and mathematical and statistical knowledge (see Figure 1).

This viewpoint is reflected also in most publications and blogs about Data Science, which claim that Data Science is unique *not in the methods or questions that are asked*, but rather in the unique combination of computational skills, statistical and domain expertise.^11^

This unique skill set enables analysts to do things differently than their traditional colleagues. It can be rightfully argued that the data deluge requires new methods, and that data scientists are well equipped to deal with the data wave just because they combine the right skills needed to acquire large amounts of data, integrate, preprocess, explore, and analyze them, interpret results, and extract knowledge from them using computational methods (Loukides, 2011). It is not easy to find scientists that are skilled in all three areas.

It can also be argued that this inherently transforms science, in the sense of a fourth paradigm (Hey et al., 2009). The new data sources are produced at an unprecedented rate, variety, and with unclear purpose and unknown quality. This requires new kinds of methods but also bears a large potential of finding new kinds of insights. For example, regarding geographic information, there have recently been so‐called “spatial turns” in Sociology (Sheller, 2017) and in health science (Cromley & McLafferty, 2011; Richardson et al., 2013) because of the new scientific potential that geodata and related methods offer for these disciplines.

However, first, the skill set that is needed for such data scientists is arguably the same that can be found in the respective disciplines, such as GIScience (to cover geodata and GIS methods), Statistics (to cover statistical methods), Machine Learning (to cover data mining methods), and Computer Science (to cover issues like data structures, databases, algorithms and complexity). Furthermore, and what is more important, skills alone do not make a discipline.

### 3.2 Data science as a community of practice

An often heard saying is that a data scientist is a better programmer than the average statistician and a better statistician than the average programmer.^12^ The skills needed for a data scientist are to be found at the intersection between Mathematics (providing structures for understanding problems and abstractions), Statistics (developing inference methods based on those structures), and Computing Science (implementing the methods, and providing systems to apply them at scale; see Figure 1).

But what about *scientific skills* and corresponding practices? Clearly, data scientists are scientists. This means that they answer questions from a particular scientific discipline. For example, geographic data scientists may be geographers, geoscientists, or health scientists. However, when answering scientific questions, a data scientist is only concerned with applying scientific methods, and not with their foundations. Data scientists precisely (and only) answer the questions within their respective empirical disciplines, such as “What is the effect of the exposure to green space on mental health?” (Roberts, Van Lissa, Hagedoorn, Kellar, & Helbich, 2019), and thereby use methods provided by another (meta‐scientific) discipline, such as Statistics, GIScience, and Machine Learning.

However, though data science mostly operates within other empirical disciplines, might it not be promising to regard it as a meta‐science discipline instead? We think there are good reasons to be sceptical about this. Though the terms “data” and “information” are often used synonymously, the important difference between them is that the latter implies selectivity and purpose (as explained in Section 2.2), which is why information is often much more costly to produce than raw data. Yet, there is nothing in the methodology of data‐driven science that could lead us to believe that the sophisticated dimensions of information, in particular, the cognitive means to turn data into information, could be handled purely through automated machine learning.^13^

We therefore argue:

Claim 4 A good data scientist is just a good scientist that makes effective use of the new data deluge.

The concepts, questions, and methods that data scientists are involved with are currently not unique to data science, as it operates within other scientific fields. And this is the main reason why data science should not be considered a science, but rather a community of practice:

Claim 5 Data Science is the community of practice of data‐driven scientists of whatever scientific discipline they ask questions about.

### 3.3 Geographic data science

Geographic Data Science answers questions in the field of Geography or related empirical disciplines that involve spatial questions. This practice covers all the stages of data‐driven geographic research: data acquisition, processing, analysis, mapping, evaluation, as highlighted using three examples in the following.

In the first example, geographic data scientists apply the LIDAR‐based digital elevation modeling method (Shan & Aparajithan, 2005) developed in GIScience to urban Geography. High‐resolution elevation data measured by laser scanners are used to generate new kinds of elevation models. By interpolating the known elevation data, we can create a DEM and thus estimate the elevation of each point of the study area. DEM is one of the important types of input data for answering many geographic questions, for example, modeling city shapes (He, 2015; Rebecca, Gold, & Kidner, 2008), wind flow (Pichugina, Banta, Brewer, Sandberg, & Hardesty, 2012), and new types of land cover classification (Yan, Shaker, & El‐Ashmawy, 2015).

The second example is from the disciplines of Environmental Science and Biology. In order to measure the distribution of species at large scale, data scientists use spatial distribution models (SDMs) to predict the probability of species occurrence based on environmental variables (Duque‐Lazo, Van Gils, Groen, & Navarro‐Cerrillo, 2016). Models are based on two types of data: data for dependent variables are presence and absence samples of a type of species (e.g., microbes, animals), data for independent variables are environmental factors of a study area (e.g., monthly temperature, soil types). Random forests and artificial neural networks are suitable machine learning methods to model living areas by training existence samples of species and environmental variables. Models predict the probability of the species occurrence for unknown areas (Duque‐Lazo et al., 2016).

Our third example illustrates that similar approaches apply also to social sciences and the humanities, in particular to Human Geography. For example, data scientists in Human Geography measure the walkability of each neighborhood of a city by calculating a walkability index in terms of residential density, intersection density, and land use mix (Van Dyck et al., 2010). Indices reflect “how friendly a city is to pedestrians,” which can then be used to investigate environmental conditions of public health (Cromley & McLafferty, 2011).

Geographic Data Science thus borrows methods and models from other disciplines in order to do Geography. For example, LIDAR technology from remote sensing, interpolation from GIScience, random forests and artificial neural networks from machine learning, and normalization from Statistics.

To summarize, Geographic Data Science (GDS) and Geographic Information Science (GIScience) are fundamentally different, even though they seem to have things in common. GDS and GIScience both deal with methods to process spatial and temporal data for supporting Geography research. This may give the impression that they are both meta‐science disciplines for Geography. However, through the discussion above, it should be clear that GDS lacks its own research questions and concepts other than the ones given by the empirical discipline. Also, GDS is inherently transdisciplinary, while GIScience constitutes a single discipline. Therefore, GDS and GIScience neither overlap nor contain each other.

## 4 WHAT IS MISSING TO BECOME A SCIENTIFIC DISCIPLINE?

A note of caution is required at this point: By saying that Geographic Data Science is currently a community of practice, we are *not* claiming that it could not become a scientific discipline. So if GDS was a discipline, what kind of discipline would it be? For this purpose, we first take a look at GIScience, which has gone through the process of becoming a scientific discipline decades ago, before we explore the potential of GDS as a discipline.

### 4.1 Geographic information science as a meta‐science discipline

The term *meta* in “meta‐science” simply means that its questions are about methods used in science, and for this very reason, meta‐science disciplines are basically *design* disciplines (Iivari, 2005). Similarly, both Statistics and Machine Learning design methodologies for representing, analyzing, and interpreting data in empirical sciences. Second, the term *science* means that questions express unique, generalizable problems, and the concepts help decompose the problem and identify its facets. For example, Statistics introduced concepts such as distribution, variance, population, and null hypothesis, to be able to distinguish data properties from a stochastic process and to be able to measure the confidence of an assumption about this process, given data.

To what extent does GIScience demonstrate the two characteristics of a meta‐science discipline? Despite the early and ongoing debate on whether GIScience should be seen merely as an engineering discipline (Reitsma, 2013), we believe there are many reasons to consider it an independent meta‐science discipline:

With respect to the *meta* part, GIScience focuses on *how* and *for what purpose* geographic data can be collected, analyzed, interpreted, and visualized (Couclelis, 2010, 2016). Numerous textbooks were written on the foundations of a theory about GIS methods and its application (Burrough, McDonnell, McDonnell, & Lloyd, 2015; Campari, 1991). For example, GIScience has developed approaches to assess fitness‐for‐purpose and data quality (Mocnik et al., 2018; Mooney, Corcoran, & Winstanley, 2010), as well as highly useful technological standards for geodata and geo‐computational processes, including the Simple Feature model (Herring, 2006) for vector geodata, the GeoSPARQL standard (Battle & Kolas, 2012) for querying geodata on the (Semantic) Web, as well as map algebra (Tomlin, 1994) for manipulating raster layers. Based on these standards, data from all over the Web can be queried and loaded into map layers, which can be combined using map algebra to derive new information for a given purpose. Data quality, query standards, map layers, and map algebra are highly specific technologies produced by GIScience and used in other disciplines. Furthermore, given the various critique raised by human geographers (Guan, Wilson, & Knowles, 2019) questioning GIS as a positivist/technology‐centric toolbox in a domain entrenched by the human condition, GIScience has responded by embracing a variety of ontological interpretations and models of vagueness for spatial information (Schuurman, 2006).

More importantly, GIScience is a *discipline* of its own right also because it defines its own *concepts* and *questions* (Burrough & Frank, 1995). We have already mentioned some of the concepts that are used at the computation level such as layers and maps. However, spatial information is not only represented in layers and maps, but it also requires to be understood and manipulated by humans (Miller, 2003). From the beginnings of the discipline, it has therefore been recognized that the concepts of spatial information are largely reflected in human cognition and language (Raubal, Mark, & Frank, 2013), for example, in the way humans perceive and reason with boundaries (Burrough & Frank, 1996; Egenhofer & Franzosa, 1991) and spatial categories (Mark, 1999), and in the way humans interpret maps (Montello, 2002) and navigate in space using spatial landmarks (Klippel et al., 2004). Furthermore, as suggested by Kuhn (2012); Kuhn and Ballatore (2015), GIS experts in practice often decompose and interpret geographic questions in terms of so‐called *core concepts of spatial information*, including location, field, object, event, and network. Since these concepts are borne in the minds of GIS experts, they can be used across data formats, software artifacts and disciplines to turn spatial questions into testable and computable answers. A *field*, for example, is a spatially continuous value surface (Kemp, 1996). Viewing the geographic world as a field not only requires a different conceptual lense, but also different computational methods, as opposed to regarding the world as a collection of objects (Couclelis, 1992). For example, to answer the question “What is the impact of Carnival on urban life?,” a Carnival procession along a road can be conceived as an event, the road the Carnival crowd follows can be regarded as a spatial network linking intersections, the Carnival crowd as an object with a spatial trajectory, the buildings surrounding the road as stationary objects, and the noise emitted by of Carnival crowd can be conceived as a field. This unique view of dividing the world into computable concepts distinguishes GIScience from other scientific disciplines. At the same time, these concepts constrain spatial analysis (Sinton, 1978) and help decompose the questions into workflows (Scheider, Meerlo, Kasalica, & Lamprecht, 2020).

Methods developed in GIScience are widely adopted in other disciplines, including Geography, Health Science, Planning, Marketing, Psychology, and Linguistics (Graves, 2008; Keenan & Jankowski, 2019; Richardson et al., 2013; Welle Donker & van Loenen, 2017). While this is certainly a positive trend, the new opportunities for data‐intensive science come with new challenges (Labrinidis & Jagadish, 2012). This raises the question of whether GIScience is ready to meet these challenges, or whether a novel discipline called Geographic Data Science may be better equipped.

### 4.2 Geographic data science as a meta‐science discipline

What could be the questions and concepts behind Geographic Data Science? As a meta‐science discipline, it should be concerned with research about data‐driven methods for geospatial or geographic questions. Since the methods are data‐driven, they need to be effective, efficient, and scalable in terms of data handling, as well as diverse in data sources and data domains. Which concepts and theories are needed for this purpose? To a large extent, these questions are already tackled by other disciplines. For example, Computer Science provides data structures and algorithms as well as memory and abstract processors. GIScience provides core concepts of spatial information, spatial formalisms, and spatial transformations as concepts. So what could be the questions and concepts that are unique for Geographic Data Science?

To understand the need for (or lack of) Geographic Data Science, we need to understand the requirements imposed by big data. Though GIScience might be well equipped to deal with the issues of scalability and diverse data sources, there are many remaining challenges (Miller & Goodchild, 2015). Consider the task of providing a universal “cyber‐infrastructure” for collaborative research (Wang, 2010), or the decades‐old vision of a digital earth (Craglia et al., 2012). Currently, GIScience does not provide a framework for handling the level of diversity and scalability of such tasks. Is there an opportunity for Geographic Data Science to carve a niche for itself as a discipline of its own? So one question, therefore, might be:

Question 1 How can data from different domains be combined, managed, analyzed, interpreted, and visualized efficiently?

Furthermore, GIScience in the past mostly focused on environments with relatively little data (Miller & Goodchild, 2015). Yet, having large amounts of diverse data can open new avenues of empirical research to answer research questions that were unanswerable before. Therefore, the focus should shift from efficiency to effectiveness in terms of offering new methods for answering previously unanswerable questions or discovering radically new solutions not envisioned by current research practices. This is the second avenue of research such a Data Science discipline could explore:

Question 2 How can big data be leveraged to tackle new problems, propose new solutions, and address existing deadlocks in research?

However, note that so far we have deliberately avoided attaching adjectives such as “spatial” or “geographic” to these questions. This means the arguments above do not support the need for *Geographic* Data Science specifically. If we focus on geographic concepts, then Geography (on the empirical level) or GIScience (on the meta‐level) will do, and if we focus on effectiveness, GIScience does a decent job in carving out spatial information purposes, qualities, and constraints. Finally, if we focus on efficiency, there is no need to reinvent the solutions developed in Computer Science. For example, regarding spatial indexing and search (Samet, 1990).

For this reason, we struggle to come up with a set of questions that would be distinctive as well as specific enough for “Geographic” Data Science.

## 5 CONCLUSION

In this article, we discussed the perspective for a new discipline referred to as Geographic Data Science. Our main conclusion is that no coherent argument can be made for requirements, research questions, and concepts that would necessitate Geographic Data Science as an independent discipline, other than a community of practice.

At the core of our argument is a distinction between an empirical and meta‐science discipline. The geographic community already has a well‐established division between GIScience, which is a meta‐science discipline about geographic information methods, and Geography as a community that addresses empirical research questions. We concluded that what is currently referred to as Geographic Data Science is a data‐driven subcommunity within Geography and Geoscience that still focuses on answering empirical questions. To become a discipline on its own, it would need to move up to the meta‐level.

Furthermore, on the meta‐level, the GDS approach lacks a range of concepts about spatial information, quality and purpose needed to deal with geodata as an object of research. This is potentially dangerous: Exchanging the label GIScience with GDS is at best naive, and in the worst case may lead to a degradation of science. While neither statisticians nor machine learning researchers would confuse their methods with their goals, the GDS approach implies that spatial information as a goal could entirely be handled in a data‐driven manner. What might be at stake is, therefore, scientific depth.

Finally, we have investigated whether there is a need for Geographic Data Science beyond a community of practice. We argued that for this purpose, Geographic Data Science needs its own set of questions and concepts that distinguish it from other disciplines, in particular from Geography and GIScience. In its current state, Geographic Data Science is not only far from satisfying this requirement, but what is required also largely matches what has been a subject of research in GIScience and other meta‐science disciplines, such as Computer Science, all along. While we have identified two major challenges that GIScience faces today, these challenges do not seem to necessitate a particular *Geographic* Data Science and can be addressed either by GIScience or within a wider effort of Data Science.

## Endnotes

1
https://community.esri.com/community/education/blog/2018/05/05/is-giscience-converging-with-data-science-should-it

2
https://www.ucgis.org/index.php?option=com_dailyplanetblog&view=entry&year=2016&month=10&day=23&id=3:space-skepticism-by-luc-anselin

3 GIScience evolved largely as the science behind Geographic Information Systems (GIS), see Goodchild (2010).

4
https://www.ucgis.org/index.php?option=com_dailyplanetblog&view=entry&year=2016&month=10&day=23&id=3:space-skepticism-by-luc-anselin

5 We should be very critical about the view that to be scientific, any science would require exposing theories to the scrutiny of as many facts whatsoever. Compare arguments in Bartley III (1968); Scheider et al. (2017); Chalmers (2013).

6 The reader may throw in *meta‐learning* (Vilalta & Drissi, 2002) as counterexample. Yet, meta‐learning is nothing more than optimising over given models and does not allow one to design a new machine‐learning algorithm.

7
http://www2.isye.gatech.edu/jeffwu/presentations/datascience.pdf

8
https://en.wikipedia.org/wiki/Data_science

9 See also https://towardsdatascience.com/is-data-science-really-a-science-9c2249ee2ce4

10
https://www.techopedia.com/definition/25827/knowledge-discovery-in-databases-kdd

11
https://towardsdatascience.com/is-data-science-really-a-science-9c2249ee2ce4

12
https://twitter.com/josh_wills/status/198093512149958656?s=21

13 See, for example, the arguments in Scheider et al. (2017). And more generally, cf. Mitchell (2019) for a clear view on the limits of most recent Deep Learning driven Artificial Intelligence.
